# MaGuS: a tool for quality assessment and scaffolding of genome assemblies with Whole Genome Profiling™ Data

**DOI:** 10.1186/s12859-016-0969-x

**Published:** 2016-03-03

**Authors:** Mohammed-Amin Madoui, Carole Dossat, Léo d’Agata, Jan van Oeveren, Edwin van der Vossen, Jean-Marc Aury

**Affiliations:** CEA, DSV, Institut de Génomique, Genoscope, 2 rue Gaston Crémieux, CP5706, 91057 Evry, France; Keygene NV, Agro Business Park 90, 6708 PW Wageningen, The Netherlands

**Keywords:** Scaffolding, Genome map, Anchoring, Whole genome profiling, Arabidopsis

## Abstract

**Background:**

Scaffolding is an essential step in the genome assembly process. Current methods based on large fragment paired-end reads or long reads allow an increase in contiguity but often lack consistency in repetitive regions, resulting in fragmented assemblies. Here, we describe a novel tool to link assemblies to a genome map to aid complex genome reconstruction by detecting assembly errors and allowing scaffold ordering and anchoring.

**Results:**

We present MaGuS (map-guided scaffolding), a modular tool that uses a draft genome assembly, a Whole Genome Profiling™ (WGP) map, and high-throughput paired-end sequencing data to estimate the quality and to enhance the contiguity of an assembly. We generated several assemblies of the Arabidopsis genome using different scaffolding programs and applied MaGuS to select the best assembly using quality metrics. Then, we used MaGuS to perform map-guided scaffolding to increase contiguity by creating new scaffold links in low-covered and highly repetitive regions where other commonly used scaffolding methods lack consistency.

**Conclusions:**

MaGuS is a powerful reference-free evaluator of assembly quality and a WGP map-guided scaffolder that is freely available at https://github.com/institut-de-genomique/MaGuS. Its use can be extended to other high-throughput sequencing data (e.g., long-read data) and also to other map data (e.g., genetic maps) to improve the quality and the contiguity of large and complex genome assemblies.

**Electronic supplementary material:**

The online version of this article (doi:10.1186/s12859-016-0969-x) contains supplementary material, which is available to authorized users.

## Background

Technical advances and cost reduction in genome sequencing have allowed the completion of numerous genome sequencing projects based on whole-genome shotgun fragments using high-throughput sequencing data and the assembly of these data. The genome assembly process usually involves four main steps: reads assembly into contiguous sequences (contigs), linking of contigs into larger gap-containing sequences (scaffolds), gap closing to fill gaps generated by the scaffolding, and anchoring onto a genetic map to build the final pseudo-molecules. During the second step, end sequences of large fragments (>1 kb) or long reads are aligned to the contigs and the alignment information is used to link contigs into scaffolds. Several commonly used scaffolding programs have been published in the last decade [1]. The efficiency of the scaffolding depends mainly on the diversity and fragment size of the input reads libraries and on the size and quality of the long reads. Typically, 1 to 20 kb libraries are used consecutively during the scaffolding step, which allows repetitive regions of various sizes to be spanned [2]. However, during the alignment step, the presence of repeated sequences creates multiple assembly solutions, which generally causes ambiguities that scaffolder programs cannot untangle. This is often the case in large and complex genomes where repetitive elements are large and cover a large fraction of the genome [3]. To decrease the number of false links, scaffolder programs require a cutoff for the minimum number of read pairs (or long reads) that validate a contigs junction; as a consequence, low-covered contigs are overlooked for scaffold building.

Access to a genome map is a great advantage in obtaining a high-quality genome assembly [4]. Genome maps can also help in detecting assembly errors by revealing discrepancies between the map and the assembly [5] and can provide independent information for evaluating genome assembly quality. Currently, several different types of genome maps can be produced to drive or improve assemblies including physical maps, optical maps, and genetic maps.

Historically, physical maps have been used for large genome sequencing projects to order clones and perform clone-by-clone sequencing, which reduces the complexity of the assembly by sequencing single or pooled clones [6, 7]. Although, this strategy is time consuming and expensive, it remains the best option for high quality genome sequencing of large and complex (polyploid) genomes such as the wheat genome [8]. Recently, the Whole Genome Profiling (WGP™) approach was developed by Keygene NV (Wageningen, The Netherlands) to create an accurate sequence-based physical map starting from a bacterial artificial chromosome (BAC) library [9]. In the WGP method, pooled BAC DNA is digested by a restriction enzyme and after amplification, Illumina technology is used to obtain sequence tags (typically 50 nucleotide sequences flanking the restriction sites). WGP has been used successfully to build physical maps of several plant genomes such as those of wheat [10] and tobacco [11].

Optical maps were used to assemble the *Amborella* [12] and goat genomes [13]. For *Amborella*, this allowed the reordering and super-scaffolding of the draft assemblies and increased their contiguity (N50 increased from 4.9 to 9.3 Mb). More recently, the release of the Irys system from BioNano Genomics provided new opportunities to improve the quality and the contiguity of genome assemblies [14].

Genetic maps allow the construction of pseudo-molecules by anchoring the assembly on linkage groups that correspond to the chromosomes [15]. Genetic map construction takes advantage of sequence-based genotyping (SBG) [16], genotyping-by-sequencing, and RAD-seq libraries [17] to obtain ultra-dense genetic linkage maps [18]. However, missing data or genotyping errors cause map inaccuracies [19]. Moreover, the physical distance between markers can be very high in genomic regions where the recombination rate is low, which makes it difficult to anchor or orientate scaffolds located in those regions.

Methods used to anchor whole-genome shotgun (WGS) assemblies on genomes have been investigated using several genetic maps to estimate assembly quality, as implemented in MetaMap [5]. The ability of these methods to produce pseudo-molecules also was tested, as reported in Popseq [20] and Allmaps [21]. Allmaps infers the sizes of gaps using the relation between the local recombination rate and the physical distance between two adjacent genetic markers; however, the estimations can be inconsistent considering the inaccuracy of the recombination rate.

Hybrid strategies, combining WGS and genome map data, are likely to help increase the quality of the assembled genome sequence. With this in mind, we developed MaGuS, a modular program that combines a genome map and WGS data. MaGuS can anchor a draft assembly onto a genome map for two applications: quality assessment of a draft assembly by calculating novel metrics, and improvement of the contiguity of a draft assembly based on evidence provided by a genome map and high-throughput screening (HTS) data. Here, we detail the MaGuS pipeline and provide an example of its applications using the Arabidopsis TAIR10 genome assembly.

## Methods

### *Arabidopsis thaliana* genome assembly

One 350-bp paired-end (PE) (ERX372154) and two 5.35-kb mate-pair (MP) (ERX372148, ERX372150) Illumina sequence libraries from *A. thaliana* were downloaded from the European Nucleotide Archive (ENA). A total of 47.6 Gb of data were obtained representing a coverage depth of 306X of PE and 91X of MP reads.

Adapters and primers were removed from the reads, and low quality nucleotides were trimmed from both ends (quality values lower than 20). Reads were also trimmed from their second N to the end and reads longer than 30 nucleotides were kept. Reads that mapped onto run quality control sequences (i.e., the PhiX genome that is used in Illumina sequencing as quality control) were removed. To decrease the number of sequencing errors present in the paired-end (PE) reads, we applied Musket v1.1 [22] with a k-mer size of 26 ‘-k 26’. We ran Kmergenie v1.5692 [23] on the PE reads to find the best k-mer size for the contig construction step and obtained an optimal k-mer size of 91 bp. SOAPdenovo2 [24] was used to perform the genome assembly, a *de Bruijn* graph was constructed with parameters ‘-K 91 –R’. As SOAPdenovo2 produces contigs over k + 1 bp, we selected informative contigs longer than 500 bp for further processing.

We used the PE and MP reads in five different scaffolding programs: SOAPdenovo2, SSPACE [25], SGA [26], BESST [27], and OPERA-LG [28]. We considered that the two main scaffolding parameters were the k-mer size used at the mapping step and the minimum number of link that validates a contig junction. To perform a scaffolding with the five scaffolders in a fair way, we chose the same parameters for the five scaffolders. We set the k-mer size to 31 bp which is more stringent than the bowtie and bwa mem default parameter, *k* = 28 and *k* = 19 respectively. We set the minimum number of link to five which corresponded to the default parameter of BESST and SSPACE. For SOAPdenovo2, we ran the *map* command with parameter ‘-k 31’, the *scaf* command with parameter ‘–L 500’, and set the minimum number of links in the configuration file as ‘pair_num_cutoff = 5’. For SSPACE, we manually set the bowtie k-mer size ‘-l 31’ and ran the program with parameter ‘-k 5’. For SGA and BESST, we first aligned the MP reads onto the contigs using BWA aln [29] with parameter ‘-l 31’. For SGA, the links file was created using the *sga-bam2de* command with parameters ‘-n 5 -m 500 --mina 31 –k 31’. The *astat* file was generated setting ‘–m 500’. The *scaf* file and the corresponding FASTA file were both created with parameters ‘–m 500’. For BESST, we chose the optimal k-mer size used for the contig assembly as ‘-K 91’ and ran the program with parameter ‘-e 5’. For each program, we selected the scaffolds that were over 2 kb in length. For OPERA-LG, we set the k-mer size for scaffolding with option ‘kmer = 91’. The minimum contig size required for the scaffolding step was fixed as 500 bp with the parameter ‘contig_size_threshold = 500’. Finally, the number of links to validate a connection between two contigs was assigned with the parameter ‘cluster_threshold = 5’.

To evaluate the quality of each assembly, we used QUAST v2.3, a popular program based on Nucmer. In the presence of a trusted reference, QUAST aligns with Nucmer the assembly to the provided reference and generates quality metrics. We observed several inconsistancies in the QUAST output. After discussions with the QUAST authors, the source code of QUAST was modified to avoid, as much as possible, the detection of misassemblies (relocation, translocation, and inversion) that correspond to false positives. Because Nucmer generated spurious alignments lower than 5 kb in highly repetitive regions, the minimum alignment length in both parts of a misassembly was set to 5 kb. Moreover, the gap or overlap size threshold length was increased to 5 kb to detect relocations. By default, QUAST reports misassemblies found within a scaffold only if at least 50 % of the scaffold is aligned. We modified this parameter to report all misassemblies regardless of the aligned fraction of a scaffold.

### Analysis of *A. thaliana* WGP data

We used the WGP data produced from the *A. thaliana col-0* BAC library by Keygene (Wageningen, The Netherlands), the method applied to generate this data is fully described by van Oeveren et al., [9]. WGP tags were ordered by an automated procedure that performed the following steps. First, fingerprinted BAC-contig data were read with BAC-contig and position information per BAC. Then, BACs were sorted on their left and right positions in the BAC-contig and assigned a rank number (identical left and right positions lead to identical ranks). Next, tag information from the WGP tag file was read and occurrences of tags per BAC were listed. For a given BAC-contig, a tag position was calculated as the mean value of BAC rank numbers on which the tag occurred. If BAC ranks were too far apart, the tag was identified as an outlier and put aside. The remaining tags were ranked according to their mean BAC rank value, possibly with equal rank scores for equal average BAC rank values.

### Map-guided scaffolding of genome using MaGuS

**Fig. 1:**
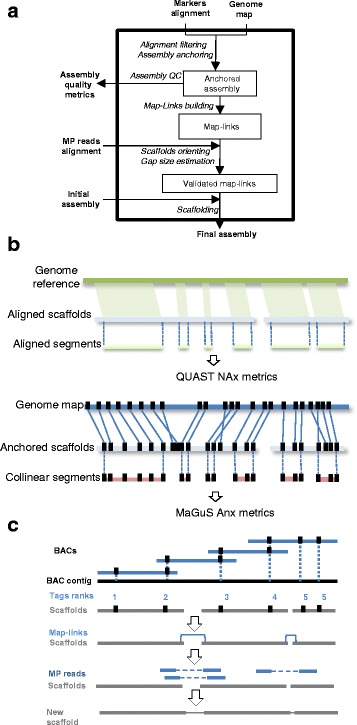
MaGuS pipeline. **a** Flowchart of the MaGuS pipeline. **b** Comparison of the QUAST and MaGuS metrics. **c** Application of MaGuS to WGP data

First, the WGP tags were aligned to scaffolds using BWA aln [29] and tags with multiple locations were filtered out of the BAM file [30]. We used the resultant alignments to anchor the scaffolds on the genome map and created links between adjacent scaffolds (Fig. 1a). However, scaffolds located within other scaffolds, according to the anchoring information, were not considered. More formally, let a mapped tag *t*(*c*, *r*) be defined by its BAC contig *c* and its rank *r* in *c*. Let a scaffold *s*((*t*_1_, *p*_1_), (*t*_2_, *p*_2_), …, (*t*_*n*_, *p*_*n*_)) be defined by the n-uplet of a (*t*_*j*_, *p*_*j*_) couple, where the tag *t*_*j*_ aligns uniquely at position *p*_*j*_ with *p*_*j*_ ≤ *p*_*j* + 1_. We define a map-link as a link between two adjacent scaffolds *s*_*i*_ and *s*_*j*_ if ${c}_{n_i}={c}_{1_j}$ and ${r}_{n_i}\le {r}_{1_j}$.

The MP reads were aligned to the assembly using BWA mem [29] and pairs whose mates mapped to different scaffolds were selected. Multiple hits were recorded and mapping possibilities that confirmed a map-link were kept. We estimated the gap size between two adjacent scaffolds using the MP fragment size distribution. If multiple scaffold orientations were reported by the read mapping, the one supported by the highest number of read pairs was selected. More formally, Let a mapping possibility of a read pair ((*scaf*_1_, *orient*_1_, *pos*_1_), (*scaf*_2_, *orient*_2_, *pos*_2_)) be defined by its scaffold name, orientation, and location of both reads with *scaf*_1_ ≠ *scaf*_2_. For each read pair, we calculate the gap size using a “naïve” approach based on the orientation of the two linked scaffolds inferred by each supporting pairs. The gap size estimation is described in (1), where μ is the mean of the MP library fragment size, *len*_1_ and *len*_2_ are the lengths of *scaf*_1_ and *scaf*_2_ respectively, *R* is the read length.

1 $$ \left\{\begin{array}{l}\begin{array}{c}\hfill ga{p}_{++}=\mu -po{s}_1-po{s}_2-2R\hfill \\ {}\hfill ga{p}_{+-}=\mu -\left(po{s}_1+R\right)-\left(le{n}_2-po{s}_2\right)\hfill \end{array}\\ {}ga{p}_{--}=\mu -\left(le{n}_1-po{s}_1\right)-\left(le{n}_2-po{s}_2\right)\\ {}ga{p}_{-+}=\mu -\left(le{n}_1-po{s}_1\right)-\left(po{s}_2+R\right)\end{array}\right. $$

We validate the link if $\mathrm{minGap}\le \frac{1}{n}{\displaystyle \sum }ga{p}_{\left( orien{t}_1, orien{t}_2\right)}$, where *n* is the number of supporting pairs for the scaffolds link with the following orientation (*orient*_1_, *orient*_2_) and minGap is the minimum gap size allowed, this value is set to −200 bp. Although, the estimated gap size proposed here has an upper bound of *μ* and may underestimate the real gap size as previously described by Sahlin et al., [31] (Additional file 1: Figure S1), the naive calculation can be used for map-link supported by one read pair which enable the validation of map-link in low covered regions. Finally, all validated links were formatted in a*.de* file for the SGA program to perform the final scaffolding, the *.de* contains the link information required by the SGA scaffolder i.e. name and orientation of the scaffolds, gap size, number of read pairs supporting the link and the standard deviation of the gap size.

### Quality evaluation of genome assembly using MaGuS

We generated new quality assembly metrics from the anchoring based on the commonly used N50 metric (used to evaluate assembly contiguity) and the NA50 introduced by the quality assessment tool QUAST (used to evaluate both contiguity and quality of assembly using a genome reference [32]). For each scaffold, we defined collinear segments as the fraction of a given scaffold that was correctly organized, i.e., segments anchored with tags that have the same order in the genome map and in the scaffolds (Fig. 1b). For a given assembly, the lengths of all these segments were used to calculate the following metrics: An50 (50 % of the anchored assembly contains collinear segments with length over An50 bp), AnA50 (50 % of the total assembly contains collinear segments with length over AnA50 bp), and AnG50 (50 % of the estimated genome size contains collinear anchored segments with length over AnG50 bp). MaGuS also generates Anx, AnAx, and AnGx graphs (based on the Nx graph [2]) that is a plot of the metrics for x values ranging from 1 to 100 %.

### Implementation of MaGuS

MaGuS was implemented in a Perl program based on five modules: *wgp2map*, which performs the anchoring and creates a MaGuS-format map that contains the anchoring information; *map2qc*, which evaluates the quality of the assembly; *map2link*, which creates the map-links between scaffolds; *pairs2links*, which validates the map-links, orients the scaffolds, estimates the gap size, and creates a link.de file; and *links2scaf*, which runs the SGA scaffolding programs and creates the final assembly.

## Results and discussion

### Arabidopsis genome assembly and quality evaluation using MaGuS

PE reads were assembled into contigs with SOAPdenovo2 (Additional file 1: Table S2). Then we generated five assemblies using five scaffolding programs (BESST, SSPACE, SOAPdenovo2, SGA, and OPERA-LG) with PE and MP reads. The BESST assembly had the highest contiguity (N50 = 1.3 Mb) followed by OPERA-LG (N50 = 1.27 Mb), SSPACE (0.98 Mb), SOAPdenovo2 (N50 = 0.82 Mb), and SGA (N50 = 0.28 Mb). To evaluate the assembly quality, we aligned the scaffolds against the Arabidopsis TAIR10 reference genome with Nucmer [33] using the QUAST pipeline [32] (see Additional file 1 for details). We found that although BESST and OPERA-LG created scaffolds that had longer alignments, they also contained relatively more misassemblies than SOAPdenovo2, SSPACE, and SGA. Based on the QUAST NA50 and NA75 metrics, we ranked the assemblies from the highest to lowest quality as BESST, OPERA-LG, SSPACE, SOAPdenovo2, and SGA.

**Fig. 2:**
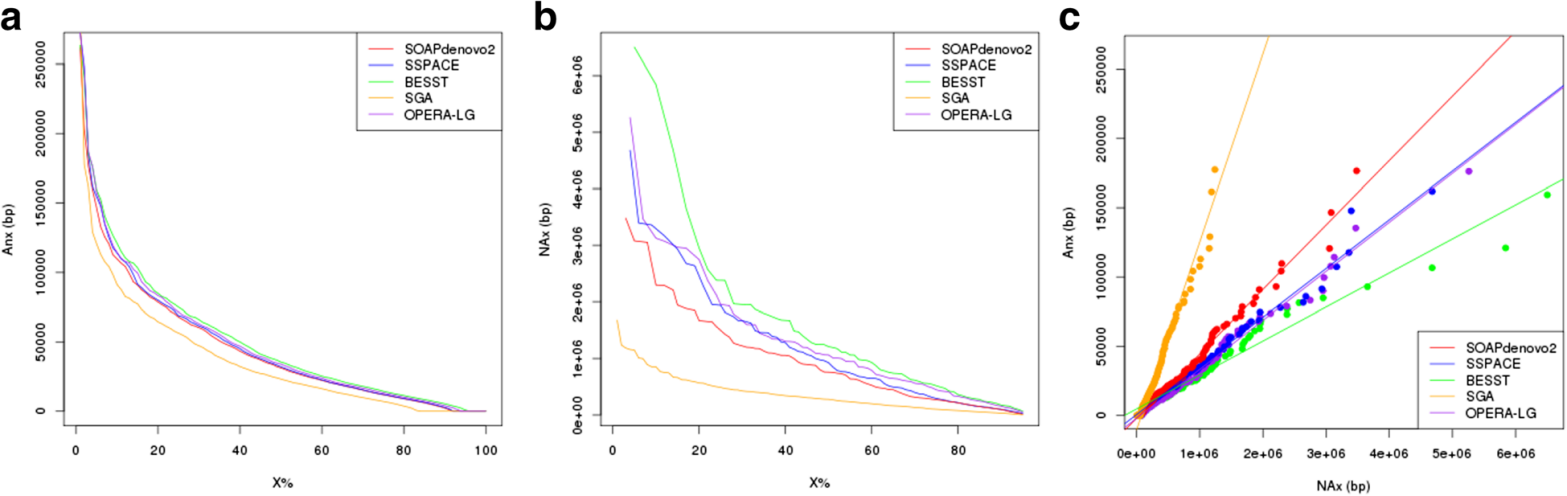
Comparison of MaGuS and QUAST quality metrics for the five assemblies. **a** MaGuS Anx plot. **b** QUAST NAx plot. **c** Correlation between Anx and NAx values

We used the WGP map to provide a reference-free approach that evaluates the quality of the five assemblies. We applied the *wgp2map* and *map2qc* modules of MaGuS to calculate the length of all collinear segments (Fig. 1b) and generated Anx values (Table 1, Fig. 2a). Considering the MaGuS An50 and the An75 metrics, the ranking of the assemblies was the same as the ranking using the QUAST NA50 and NA75 metrics. The NAx and Anx values were strongly correlated (*R*^*2*^ > 0.96) for the five assemblies (Fig. 2c), which allowed us to consider using the MaGuS Anx metrics to compare assembly quality.

**Table 1:** QUAST and MaGuS quality metrics for the five assemblies. The *R* ^2^ values indicate the Pearson correlation coefficients between the QUAST NAx and MaGuS Anx values

Assembly metrics	SOAP	SSPACE	SGA	BESST	OPERA_LG
Assembly size (bp)	115 319 220	116 017 208	114 956 386	114 996 281	116 406 702
N50 (bp)	821 817	982 887	284 070	1 299 606	1 272 891
L50	39	31	115	22	26
N75 (bp)	306 051	340 070	118 727	643 037	566 836
L75	96	81	270	54	60
QUAST metrics					
Number of N's per 100 kb	3851.60	3000.11	4251.16	2845.19	3139.94
Misassemblies	9	9	3	23	51
Largest alignment (bp)	3 482 036	4 678 885	1 680 656	6 501 653	5 259 610
NA50 (bp)	757 250	926 429	276 557	1 210 586	945 419
NA75 (bp)	268 694	291 099	100 235	516 026	351 844
MaGuS metrics					
An50 (bp)	31 217	32 028	23 164	35 466	33 908
An75 (bp)	11 887	12 052	6 981	14 315	13 113
*R* ^2^	0.99	0.98	0.96	0.99	0.96

Selecting the appropriate bioinformatics tools to perform genome de novo assembly is difficult and often depends on the genome complexity and on the sequencing technology used. The absence of a reference sequence leads automatically to the selection of the assembly that has the highest contiguity with no regards to the quality. In the present case, access to a genome map and its use with MaGuS allowed the BESST assembly to be selected as being the most continuous and also the most collinear to the WGP map.

### Arabidopsis genome map-guided scaffolding using MaGuS

**Fig. 3:**
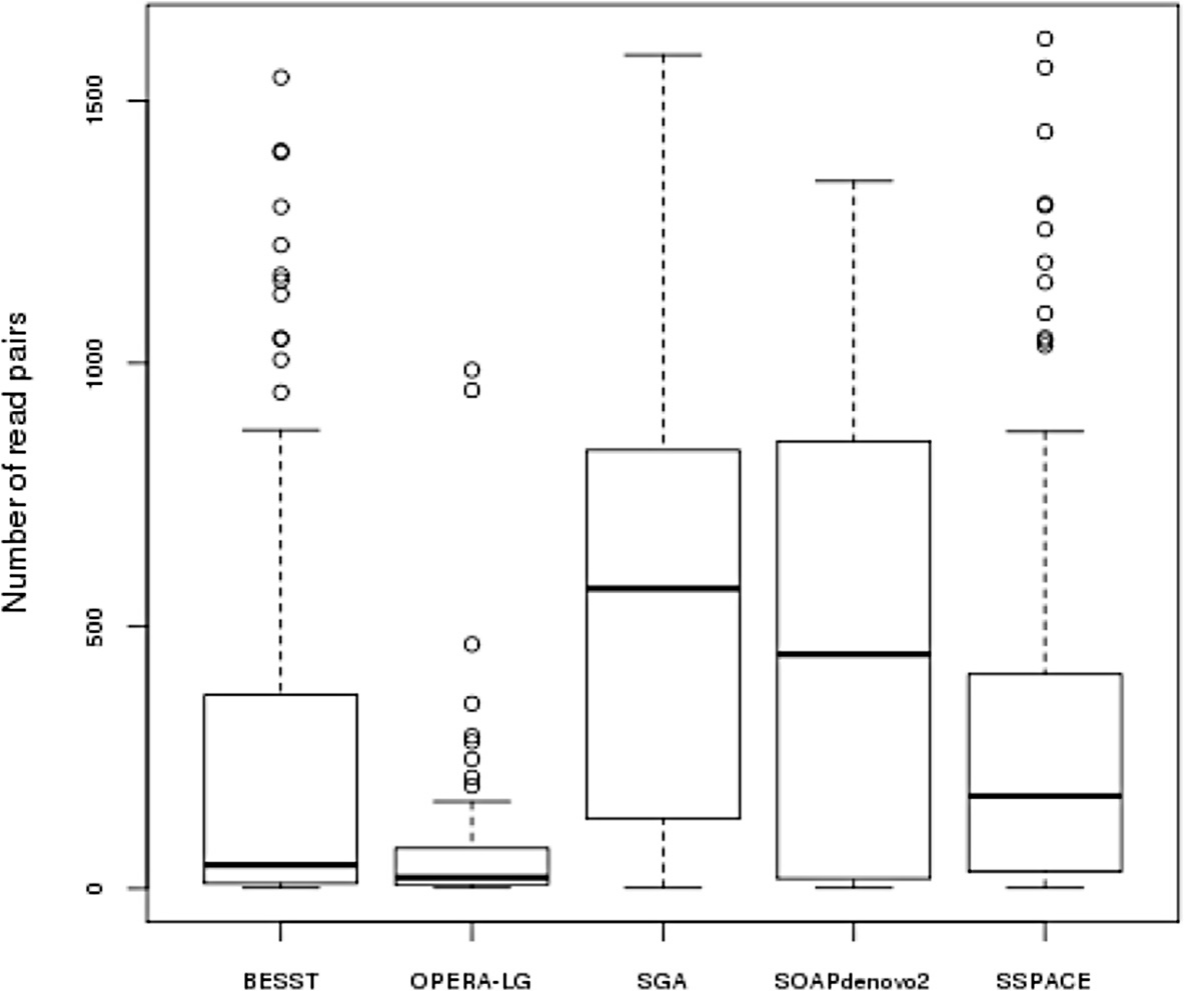
Distribution of the number of mate-pairs that validates map-links for the five assemblies

We used the five assemblies produced previously to perform map-guided scaffolding through the MaGuS pipeline (Fig. 1c). For each assembly, we first created the map-links (i.e., the links between two adjacent anchored scaffolds) and aligned the MP reads onto the scaffolds to validate the map-links by first determining the scaffolds orientation (if the scaffold was anchored by only one tag) and then by estimating the new gaps size (see Methods). The validated map-links were used to build the final scaffolds (Table 2). Only a fraction of the map-links (21.2 to 49.9 %) was validated by the MP reads. This limitation was clearly due to the MP library size, and a higher fraction of map-links would certainly be validated using larger MP libraries. Although only a fraction of the map-links were used for the scaffolding, the resulting assemblies showed increases in the N50 metrics ranging from 1.13 to 2.24-fold and increases in N75 from 1.23 to 2.43-fold (Table 2). To evaluate the accuracy of this scaffolding approach, we aligned the five assemblies generated by MaGuS onto the Arabidopsis TAIR10 reference genome using QUAST. MaGuS generated 86 % to 97 % correct links for the five assemblies and only a limited number of misassemblies (Table 2). The quality of the scaffolds also was confirmed by elevated NA50 and NA75 values. The number of read pairs that validated a map-link had a very wide distribution, from 1 to over 1000 read pairs (Fig. 3), which showed that MaGuS enabled the scaffolding of both low covered and highly covered regions that corresponded to repetitive regions.

**Table 2:** Assembly metrics after MaGuS scaffolding for the five assemblies

	SOAP	SSPACE	SGA	BESST	OPERA-LG
Assembly size (bp)	115 563 956	116 414 299	115 703 449	115 174 685	116 556 828
N50 (bp)	1 350 715	1 680 424	635 106	1 751 177	1 442 963
N50 fold change	1.64	1.74	2.24	1.35	1.13
L50	23	18	47	18	22
N75 (bp)	509 384	646 442	288 240	787 050	695 198
N75 fold change	1.66	1.9	2.43	1.22	1.23
L75	58	48	110	42	51
Number of N's per 100 kb	4 055.34	3 331.14	4 869.38	2 995.68	3 264.70
Largest alignment	5 012 555	7 708 756	3 361 051	6 902 343	5 597 743
NA50	1 187 620	1 455 792	579 394	1 407 579	1 258 868
NA50 fold change	1.57	1.57	2.1	1.16	1.18
NA75	354 088	508 625	215 751	609 320	560 902
NA75 fold change	1.32	1.75	2.15	1.18	1.59
Total misassemblies	23	19	19	36	62
Magus misassemblies	14	10	16	13	5
Number of map-links	534	481	1 034	371	368
Number of MP-validated links	209 (39.14 %)	214 (44.49 %)	516 (49.9 %)	93 (25.07 %)	78 (21.2 %)
Number of correct MP-validated links	195 (36.51 %)	204 (42.41 %)	500 (48.53 %)	80 (21.56 %)	73 (19.83 %)
False positive rate	6.7	4.7	3.1	14	6.4

### Effect of genome map errors on the MaGuS performance

To investigate the different types and levels of errors present in the Arabidopsis WGP map, we first aligned the WGP tags on the TAIR10 reference and selected the tags aligning at a single location. We defined the genomic positions of each BAC contig on the chromosomes and compared the tag rank on the WGP map to their rank inferred from their position on the assembly. This allowed us to define two types of error. The first error type concerned tags that have a different rank on the assembly but that are located within its BAC contig genomic location. The second error concerned tags that have a genomic position flanked by tags belonging to another BAC contig. Among the 54 990 tags that mapped on TAIR10 at a single position, 89.5 % had a WGP map rank that was compatible to their genomic position, 6.15 % tags were misplaced within the same BAC contig and 4.4 % were placed in another BAC contig.

To determine the effect of the two error types on the MaGuS performance, we simulated artificial WGP maps that contained different error levels. We first generated an error-free WGP that contained 191 BAC contigs and 64 441 tags. Within a BAC contig, the occurrence of each rank was set randomly from a gamma distribution (shape = 1.07 and scale = 0.16), the parameters were inferred from the rank occurrence observed in the Arabidopsis WGP data. For the two error types, we created WGP maps by adding random errors on the error-free WGP map. Errors were added with the following rates: 0.01, 0.05, 0.1, 0.2, 0.5 and 0.8. For each error type and error rate value, five random maps were generated as replicates. A total of 60 simulated maps (2 error types × 5 error rates × 5 replicates = 60 simulated maps) were generated.

**Fig. 4:**
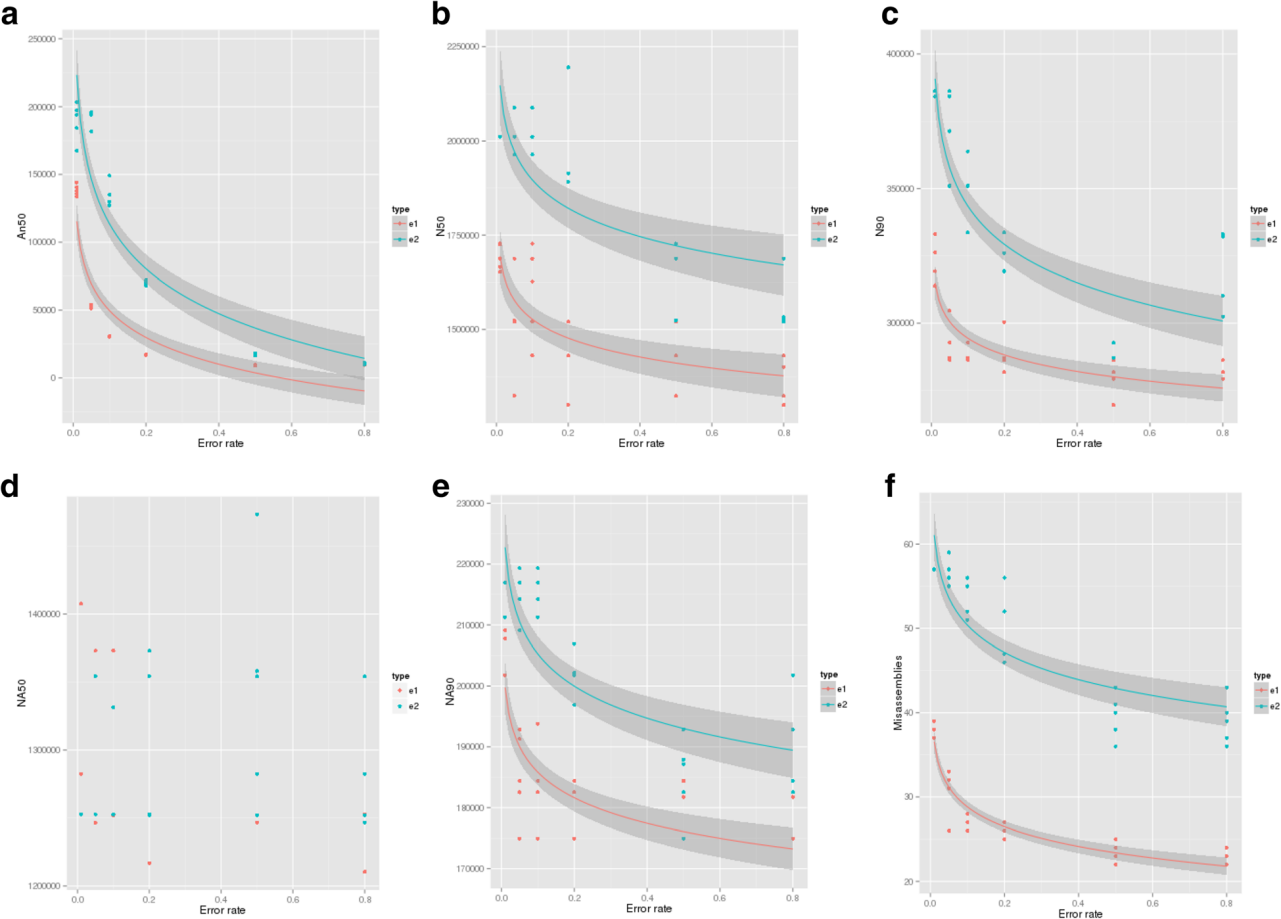
Effect of intra and inter-BAC contig errors on MaGuS scaffolds. The intra and inter-BAC contig errors are named e1 and e2 respectively. **a**. Effect of the map errors on the An50 values. **b**. Effect of the map errors on the N50. **c**. Effect of the map errors on the N90. **d**. Effect of the map errors on the NA50. **e**. Effect of the map errors on the NA90. **f**. Effect of the map errors on misassemblies. Grey areas are values between the upper and lower pointwise confidence interval around the mean, these values were obtained from a log regression

We ran the MaGuS pipeline using the simulated maps, the assembly produced by the BESST scaffolder and the mate-pair reads. The effect of the errors to the collinearity between the TAIR10 sequence and the simulated WGP maps is represented by the variation of the An50 values on Fig. 4a. The An50 values decreased for error rates over 0.1 which was expected and validates the construction of the simulated WGP maps. The N50 and N90 values of the MaGuS scaffolds were impacted by the intra and inter-BAC contig errors (Fig. 4b and c). We compared the N50 obtained for each error rate and found no significant differences between N50 for the intra-BAC contig error rate of 0.01, 0.05 and 0.1 (Tukey test *p*-value adjusted > 0.05), the first significant changes in N50 was obtained for intra-BAC contig error rate = 0.2 (Tukey test p-value adjusted = 0.02) and for intra-BAC contig error rate = 0.5 (Tukey test p-value adjusted = 0.00009). The quality of the MaGuS scaffolds was analysed using QUAST on the TAIR10 sequence reference. Whereas the NA50 values were not impacted by the errors added in the artificial maps (Fig. 4d and e), the NA90 values were affected by both error types. The amount of misassemblies found in the scaffolds was higher for those generated from the map containing inter-BAC contig errors than for those generated from the map containing intra-BAC contig errors (Fig. 4f). We also noted that fewer misassemblies occurred when the error rate increases which can be explained by the fact that less junction were found to be validated by the mate-pair reads. The simulation of artificial WGP maps containing intra and inter–BAC contig errors at different rates showed that the map-guided scaffolding accuracy is not affected by errors whereas the contiguity depends closely on the noise of the WGP map (for intra as well as inter-BAC contig error rate over 0.1). As the WGP data of Arabidopsis have an intra and inter-BAC contig error rate under 0.1, we can consider that the Arabidopsis WGP dataset was clean enough to be efficiently used by MaGuS for a guided scaffolding.

### Effect of the input assembly contiguity on the MaGuS performance

To assess the impact of the assembly fragmentation on the magus performance, we generated eight Arabidopsis genome assemblies using different depths of coverage (10, 20, 50, 100, 150, 200, 250 and 300X of PE reads). Each read set was assembled using SOAPdenovo2 with the same parameters used in the previous section but for the k-mer size which was inferred by Kmergenie. The contigs were scaffolded with the MP reads by SSPACEv2. The resulting scaffolds were used by MaGuS to perform the map-guided scaffolding by integrating the WGP map and the MP reads (Additional file 1: Table S3). For the assemblies based on the 10× and 20× read sets MaGuS weakly improved the contiguity of the scaffolds with a N50 MaGuS scaffolds/N50 scaffolds ratio (<1.07). For assemblies based on read sets built with 50× or more, we observed an improvement of the N50 MaGuS scaffolds/N50 scaffolds ratio (>1.2).

## Conclusions

The method presented here and implemented in MaGuS enabled the evaluation of the quality and the scaffolding of a draft genome assembly using a physical map and HTS data. Its application to Arabidopsis with a WGP map provides a first example of its efficiency in reconstructing a eukaryotic genome. Evaluating the quality of a genome assembly is necessary in order to increase the accuracy of downstream analyses, such as genome annotation or comparative genomic analyses. De novo assembly projects often lack a genome reference and different ways to assess the assembly quality have been investigated [2, 34] using either the HTS data used for the assembly or a genome map. The latter remains a very good independent source of information for this task. From this perspective, we developed the *map2qc* module of MaGuS to provide assembly quality metrics. Its application to five Arabidopsis genome assemblies showed that the new quality metrics based on the correctly anchored segments of the assembly gave the same assembly ranking as if a reference genome was available.

Existing scaffolder tools encounter issues when dealing with repeat-rich regions. The use of a map overcomes this problem if a contig or scaffold can be anchored onto the map. For large genomes, the sequencing depth of an MP library may result in low covered regions. Users of scaffolding programs often set a minimum cut-off for read pairs required to validate a link between contigs, to avoid assembly errors. The use of a map to guide the assembly allows this cut-off to be lowered without loss of accuracy. The use of MaGuS is not restricted to WGP maps, other genome map types can be integrated after formatting. For example, genetic maps can be provided as input, however the performance will greatly depend on the marker density.

### Availability of supporting data

Arabidopsis Illumina reads can be downloaded from the European Nucleotide Archive (ENA) with the following IDs: ERX372154, ERX372148, ERX372150. The WGP data and MaGuS can be accessed through GitHub at https://github.com/institut-de-genomique/MaGuS.

## Additional file

Additional file 1: The supporting data are included as a single additional file which contains **Figure S1**, **Table S2** and **Table S3.** (DOCX 38 kb)
