# Supplementary material for: MaGuS: a tool for quality assessment and scaffolding of genome assemblies with Whole Genome Profiling™ Data
Source: BMC Bioinformatics. 2016 Mar 3;17:115. doi: 10.1186/s12859-016-0969-x (PMC4776351; doi:10.1186/s12859-016-0969-x)
Supplement: Additional file 1: — The supporting data are included as a single additional file which contains Figure S1, Table S2 and Table S3. (DOCX 38 kb) [file 12859_2016_969_MOESM1_ESM.docx]

**MaGuS: a tool for quality assessment and scaffolding of genome assemblies with Whole Genome Profiling™ Data**

**Mohammed-Amin Madoui^1^**^§^**, Carole Dossat^1^, Léo d’Agata^1^,** [**Jan van Oeveren**](http://www.ncbi.nlm.nih.gov/pubmed/?term=van%20Oeveren%20J%5Bauth%5D)**^2^, Edwin van der Vossen^2^, Jean-Marc Aury^1^**

^1^CEA, DSV, Institut de Génomique, Genoscope, 2 rue Gaston Crémieux, CP5706, 91057 Evry, France

^2^Keygene NV, Agro Business Park 90, 6708 PW, Wageningen, The Netherlands

^§^Corresponding author

**
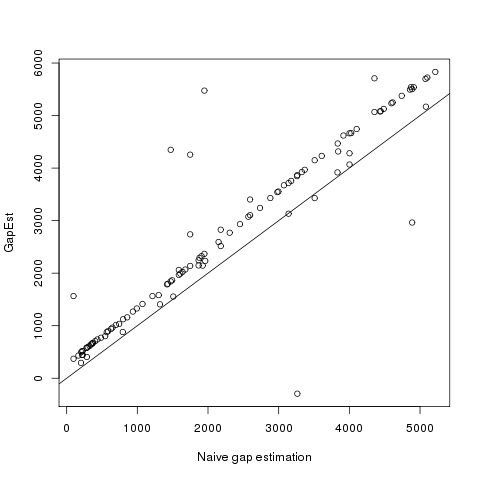
**

**Figure S1: Difference between the naïve and the gapest gap size estimation**

The gap size estimation based on the naïve approach is plotted on the x axis and the gap size estimation based on gapest is plotted on the y axis. The black line corresponds to y=x.

**Table S2: Assembly metrics of soapdenovo2 contigs**

| Assembly size | 113 253 279 |
| --- | --- |
| maxSize | 101 088 |
| minSize | 500 |
| nb contigs | 17 526 |
| averageSize | 6462.02 |
| N50 | 13 736 |
| L50 | 2 405 |
| N80 | 5 610 |
| L80 | 6 205 |
| N90 | 3 111 |
| L90 | 8 866 |

# .

# Table S3: Effect of the input assembly contiguity on the MaGuS performance.

| Coverage | 10X | 20X | 50X | 100X | 150X | 200X | 250X | 300X | |
| --- | --- | --- | --- | --- | --- | --- | --- | --- | --- |
| kmer size contig | 21 | 41 | 65 | 71 | 75 | 87 | 89 | 91 |  |
| CONTIGS METRICS |  |  |  |  |  |  |  |  |  |
| Assembly size | 90 315 709 | 105 040 711 | 110 641 941 | 111 504 136 | 111 946 200 | 113 001 125 | 113 125 776 | 113 253 578 |  |
| maxSize | 18 787 | 82 133 | 115 412 | 138 568 | 148 264 | 133 468 | 101 508 | 101 088 |  |
| minSize | 500 | 500 | 500 | 500 | 500 | 500 | 500 | 500 |  |
| nb contigs | **50 699** | **23 376** | **18 194** | **15 861** | **15 680** | **17 000** | **17 107** | **17 692** |  |
| averageSize | 1781.41 | 4493.53 | 6081.2 | 7030.08 | 7139.4 | 6647.1 | 6612.8 | 6401.4 |  |
| N50 | **2 396** | **8 083** | **12 805** | **15 972** | **16 300** | **14 250** | **14 117** | **13 517** |  |
| L50 | 11 390 | 3 793 | 2 539 | 2 002 | 1 997 | 2 307 | 2 330 | 2 442 |  |
| N80 | **1 167** | **3 438** | **5 115** | **6 315** | **6 457** | **5 770** | **5 729** | **5 504** |  |
| L80 | 27 580 | 9 707 | 6 543 | 5 235 | 5 192 | 5 944 | 6 015 | 6 296 |  |
| N90 | **825** | **2 022** | **2 842** | **3 414** | **3 474** | **3 200** | **3 186** | **3 071** |  |
| L90 | 36 765 | 13 623 | 9 374 | 7 599 | 7 518 | 8 532 | 8 613 | 8 988 |  |
| SCAFFOLDS METRICS |  |  |  |  |  |  |  |  |  |
| Assembly size | 97 432 429 | 108 740 346 | 112 909 546 | 113 646 931 | 113 963 152 | 114 833 478 | 114 915 041 | 114 977 744 |  |
| maxSize | 2 581 155 | 7 788 802 | 5 866 514 | 5 761 324 | 6 637 079 | 5 417 589 | 4 596 250 | 8 390 745 |  |
| minSize | 2 000 | 2 004 | 2 001 | 2 003 | 2 000 | 2 008 | 2 024 | 2 016 |  |
| nb scaff | 2 179 | 897 | 678 | 598 | 581 | 445 | 457 | 411 |  |
| averageSize | 44714.3 | 121 227 | 166 533 | 190 045 | 196 150 | 258 053 | 251 455 | 279 751 |  |
| N50 | **923 138** | **1 267 089** | **966 232** | **1 291 718** | **1 359 213** | **1 412 223** | **1 388 386** | **1 399 886** |  |
| L50 | 35 | 22 | 30 | 23 | 21 | 24 | 27 | 21 |  |
| N80 | **311 579** | **321 611** | **305 422** | **362 501** | **364 854** | **410 766** | **429 294** | **485 539** |  |
| L80 | 90 | 73 | 89 | 70 | 68 | 73 | 74 | 63 |  |
| N90 | **76 500** | **151 410** | **172 605** | **165 346** | **188 002** | **243 359** | **248 346** | **255 485** |  |
| L90 | 147 | 119 | 138 | 116 | 110 | 110 | 109 | 96 |  |
| MAGUS SCAFFOLDS METRICS |  |  |  |  |  |  |  |  |  |
| Assembly size | 97 646 876 | 108 885 429 | 113 068 639 | 113 844 181 | 114 143 070 | 115 025 305 | 115 070 492 | 115 138 638 |  |
| maxSize | 4 238 706 | 7 788 802 | 5 866 514 | 5 761 324 | 7 250 153 | 6 091 343 | 7 069 507 | 8 638 720 |  |
| minSize | 2 000 | 2 004 | 2 001 | 2 003 | 2 000 | 2 008 | 2 024 | 2 016 |  |
| nb scaff | 2 081 | 819 | 594 | 507 | 493 | 357 | 380 | 332 |  |
| averageSize | 46923.1 | 132 949 | 190 351 | 224 545 | 231 528 | 322 200 | 302 817 | 346 803 |  |
| N50 | **971 740** | **1 344 096** | **1 239 668** | **1 748 235** | **1 894 462** | **1 603 557** | **1 670 218** | **1 750 595** |  |
| L50 | 32 | 20 | 24 | 19 | 18 | 21 | 20 | 18 |  |
| N80 | **329 307** | **371 743** | **364 771** | **446 346** | **452 252** | **567 293** | **542 770** | **683 983** |  |
| L80 | 85 | 65 | 75 | 60 | 55 | 58 | 57 | 50 |  |
| N90 | **82 869** | **165 373** | **190 201** | **221 624** | **266 756** | **342 113** | **314 372** | **343 523** |  |
| L90 | 140 | 108 | 117 | 96 | 88 | 84 | 84 | 72 |  |
| An50 | **26 129** | **31 878** | **32 236** | **32 533** | **33 902** | **34 338** | **34 657** | **36 006** |  |
| An75 | 11 444 | 13 184 | 12 510 | 12 907 | 13 353 | 13 632 | 13 832 | 14 315 |  |
| An90 | **3 244** | **4 292** | **3 583** | **3 775** | **4 322** | **4 587** | **4 677** | **5 358** |  |
| AnA50 | 3 176 | 10 298 | 10 145 | 10 959 | 11 683 | 11 987 | 12 042 | 12 750 |  |
|  |  |  |  |  |  |  |  |  |  |
| AnA75 | 0 | 0 | 0 | 0 | 0 | 0 | 0 | 0 |  |
| AnA90 | 0 | 0 | 0 | 0 | 0 | 0 | 0 | 0 |  |
| AnG50 | **0** | **5 632** | **7 611** | **8 627** | **9 417** | **10 057** | **10 079** | **10 930** |  |
| AnG75 | 0 | 0 | 0 | 0 | 0 | 0 | 0 | 0 |  |
| AnG90 | 0 | 0 | 0 | 0 | 0 | 0 | 0 | 0 |  |
